# TOGA Therapeutic Oxygen for Gastrointestinal Atony

**DOI:** 10.1016/j.gastha.2023.12.013

**Published:** 2024-01-03

**Authors:** Brian Weiner, Chris Forsmark, Vikas Khular, Alexandra Bauman, Selina Sutchu, Debdeep Banerjee, Donevan Westerveld, Wei Zhang, Max Jacobson, Joseph Grajo

**Affiliations:** 1 Division of Gastroenterology, Bruce W. Carter Veterans Administration Hospital, Miami, Florida; 2 Medicine/Gastroenterology, Florida Atlantic University Schmidt College of Medicine, Boca Raton, Florida; 3 Division of Gastroenterology, Department of Medicine, University of Florida College of Medicine, Gainesville, Florida; 4 Department of Medicine, University of Florida College of Medicine, Gainesville, Florida; 5 Department of Radiology, University of Florida College of Medicine. Gainesville, Florida

**Keywords:** Ileus, Small Bowel Obstruction, Ogilvie’s Syndrome, Oxygen, TOGA

## Abstract

**Background and Aims:**

Ileus, mechanical bowel obstruction, and acute colonic pseudo-obstruction are characterized by distension of the intestines with accumulated bowel gas. Current treatments are not completely satisfactory.

**Methods:**

By manipulating the partial pressures of oxygen and nitrogen in the trapped air with a novel 6-hour treatment with 100% oxygen via nonrebreather mask, the bowel can be successfully decompressed, facilitating resolution of the underlying condition.

**Results:**

A positive clinical response was seen in 7/8 [87.5%] patients after therapeutic oxygen for gastrointestinal atony. Maximal lumen diameter decreased by an average of 1.14 ± 0.87 cm [16%].

**Conclusion:**

In this first clinical report of therapeutic oxygen for gastrointestinal atony, the provision of 100% oxygen via nonrebreather mask is a useful therapy. It decreased the diameter of the intestinal lumen and enhanced resolution of ileus, acute colonic pseudo-obstruction, and bowel obstruction. This is a low-morbidity, low-cost treatment of gastrointestinal luminal distension.

ClinicalTrials.gov Identifier NCT03386136.st.

## Introduction

Oxygen via non rebreather therapy [NRB] is a long-established treatment for hypoxemia. It is rarely used ‘off-label’ as a treatment for other ailments where gas exchange may play a role. Inhalation of oxygen manipulates the relative compositions of gases entering the lung, dissolved in the blood, and then undergoing exchange across the wall of the gastrointestinal tract.

**Figure 1:**
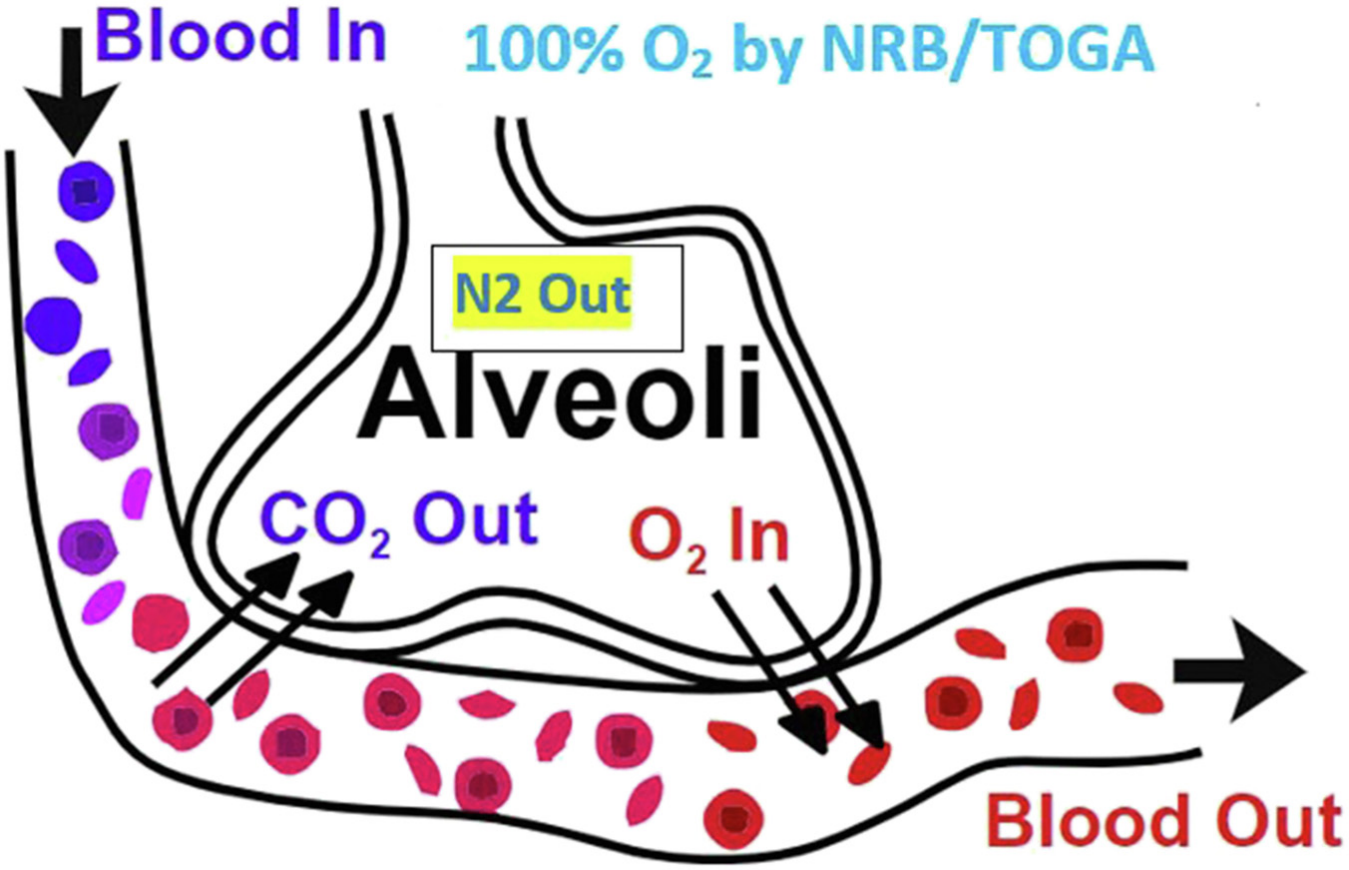
Schematic representation of an alveolus of the lung in a patient receiving 100% oxygen by nonrebreather mask. Essentially, all nitrogen has been flushed from the inspired gases. This creates a gradient between nitrogen dissolved in plasma and the alveolus. Nitrogen goes down its gradient into the alveolus and is vented into the atmosphere.^3^

While breathing room air at sea level, normal total gas pressure in the end capillary is 706 mmHg. This decrease from atmospheric pressure, 760 mmHg, is consequent to decreased partial pressure of oxygen from 100 mmHg to 40 mmHg from the arterial to the venous end of the capillaries. When breathing 100% oxygen, the total end-capillary gas tension drops to 146.5 mmHg. This is due to the absence of nitrogen, which has a partial pressure of 573 mmHg in the normal capillary (Figure 1).^1^ Northfield postulated that this nitrogen gradient and pressure gradient across the capillary bed would increase the rate at which air in the pneumothorax is absorbed. The fall in partial pressure of nitrogen in the blood accelerates its diffusion from the pleural space into the blood. On room air, pneumothorax volume is reabsorbed at a rate of 1.25% per day. On high-flow oxygen by NRB, the volume of the pneumothorax decreased at a rate four-fold that of normal.^2^

**Figure 2:**
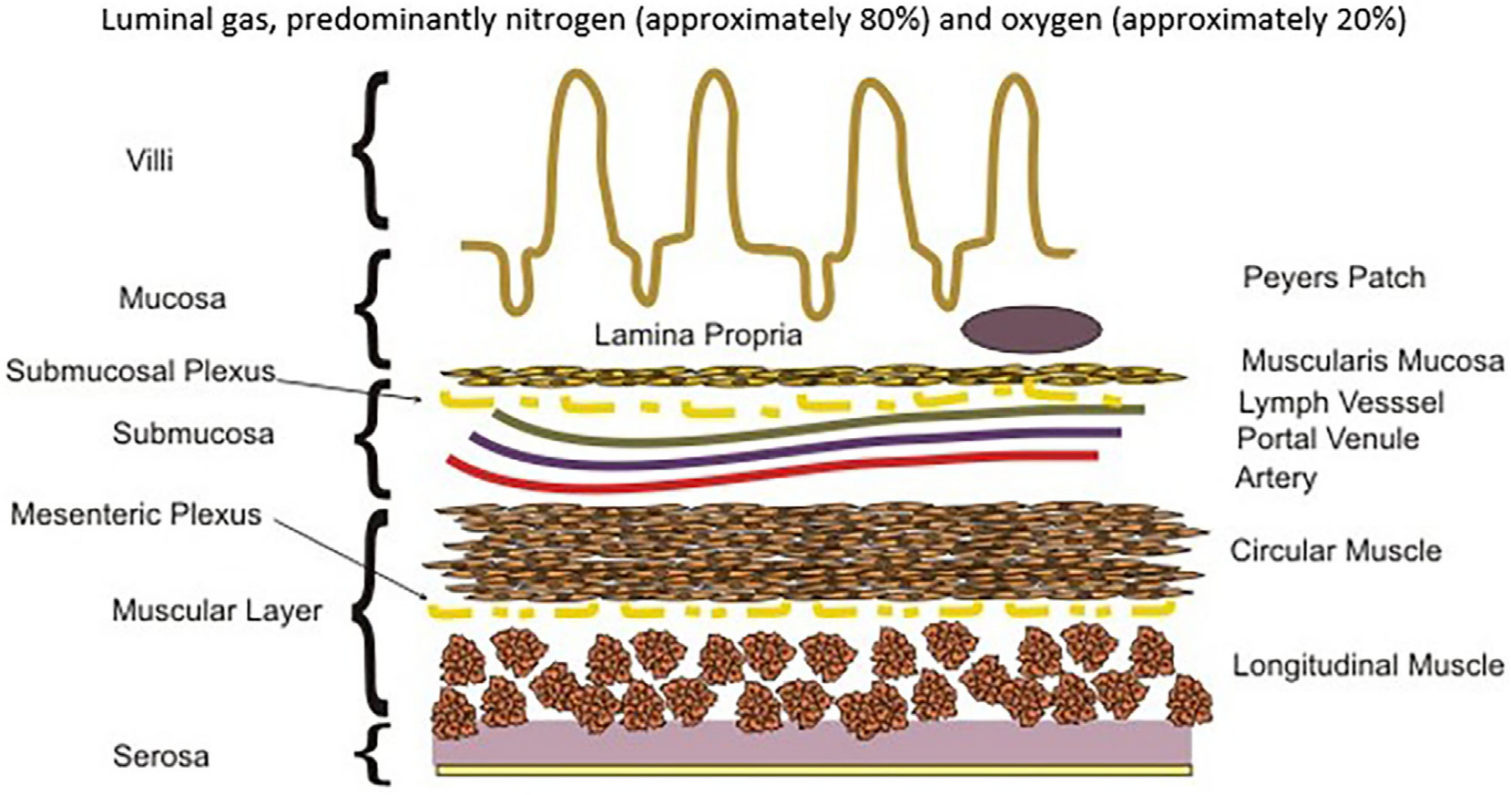
Cross-sectional image of a representative portion of the gut wall. The lumen of the bowel (top) contains predominantly atmospheric air. This is approximately 80% nitrogen. The gut wall is well perfused with capillaries in the submucosa. The plasma is essentially free of nitrogen. Nitrogen diffuses down its gradient, from the intestinal lumen to the capillary blood. This blood returns to the lungs and releases nitrogen into the alveolus.^7^

**Figure 3:**
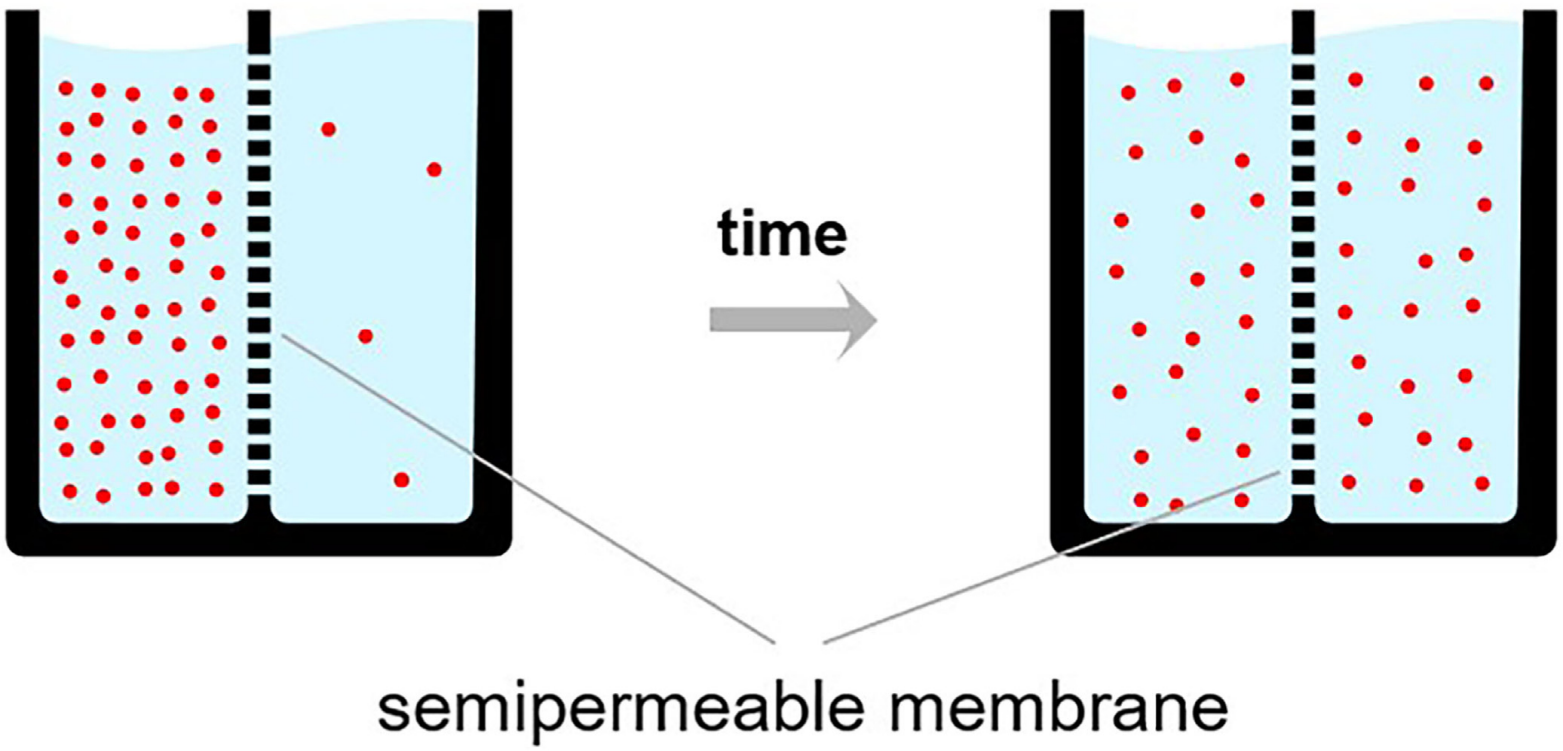
Illustration of the principle of diffusion of molecules across a semipermeable membrane. In these containers, the left-side compartment is separated from the right-side compartment by a semipermeable membrane. In the lung/alveolus situation, a high concentration of nitrogen, indicated by the orange dots, leaves plasma for the alveolus. This will be vented to the atmosphere by respiration. In the gut, a high concentration of nitrogen goes down its gradient to the plasma perfusing the bowel wall. This nitrogen is expired, and the cycle repeats during TOGA therapy.^8^

We postulated that a similar pressure gradient in the capillaries of the mesentery would produce a similar effect of gas absorption from a pathologically distended bowel. In the 1940s, Dr Kirk collected rectal gas specimens and found that the average gas composition was 59% nitrogen, 9% carbon dioxide, 3.9% oxygen, 7.2% methane, 20.9% hydrogen, and 0.00028% hydrogen sulfide. It is known that both hydrogen and sulfur hexafluoride (SF_6_) gas are absorbed across the wall of the intestines by mesenteric capillaries. These gases were measurable in expired lung air.^4^, ^5^, ^6^ If a water-insoluble gas such as sulfur hexafluoride was able to be partially absorbed and expired, then it is reasonable to hypothesize that the other bowel gas components such as nitrogen, carbon dioxide, and methane can be absorbed as well. Therapeutic oxygen for gastrointestinal atony (TOGA) is postulated to drive dissolved nitrogen out of the blood perfusing the lung, creating a nitrogen gradient between the blood and the lumen of the bowel. This nitrogen gradient will facilitate the removal of gaseous distension from the bowel lumen and accelerate resolution of ileus and bowel obstruction (Figures 2 and 3).

## Materials and Methods

The TOGA intervention involves treatment of ileus, pseudo-obstruction, or obstruction with 100% oxygen via NRB for 6 hours. The protocol offered the possibility of repeat therapy once if symptoms did not resolve. Prior to the initiation of this trial, several patients were treated with TOGA with standard clinically informed consent. In this prospective pilot series, we anticipated enrolling up to 20 patients diagnosed with ileus, small bowel obstruction, or Ogilvie’s syndrome. This trial was intended to demonstrate the feasibility, utility, and safety of TOGA.

Subjects were recruited by the gastroenterology consult service at the Shands Hospital, University of Florida, Gainesville, FL, between March and May 2018. To avoid trivial results, subjects had to have ileus or bowel obstruction for at least 24 hours and not be responsive to therapies provided by the patient’s clinical team. For each subject, the study terminated: a. after either resolution of ileus or bowel obstruction; b. failure of TOGA as indicated by worsening ileus and requirement for surgical or endoscopic intervention; or c. failure of therapy as indicated by lack of clinical and radiologic response to 2 episodes of TOGA therapy. TOGA could be discontinued if there was an adverse reaction to therapy. The investigators could recommend a second treatment with TOGA no earlier than 24 hours after the initiation of the first TOGA treatment if the results of the first TOGA session were equivocal or did not provide adequate relief for the condition under consideration. Inclusion criteria are in Table 1. Exclusion criteria are in Table 2. Diagnostic criteria are in Table 3.

**Table 1:** Inclusion Criteria

• Age 18 or older. • Informed consent obtained. • Hospitalized inpatient diagnosed with ileus, bowel obstruction, or colonic pseudo-obstruction [clinician interpretation or small bowel diameter ≥3.5 cm, cecal diameter ≥ 9 cm, sigmoid colon diameter ≥ 6 cm]. • Clinically and hemodynamically stable. • Supplemental oxygen greater than 2 L/min not required. • No contraindications to 100% oxygen. • Failure to respond to nonsurgical/nonendoscopic therapy for at least 24 h. • TOGA may be given in conjunction with: ○ Decompression with nasogastric tube and/or rectal tube ○ IV fluids and correction of volume and electrolyte status ○ NPO ○ Neostigmine ○ Proton pump inhibitor and/or histamine 2 blocker ○ Enema/laxative

**Table 2:** Exclusion Criteria

• Less than 18 y old. • Not expected to survive in short term. • Pregnant or lactating. • Severe or unstable psychiatric disorders. • Participation in concomitant research studies that would interfere with this study. • Alcohol or drug abuse. • Respirator support required. • Unable to tolerate 100% oxygen. • Perforated viscus. • Inability to obtain informed consent. • Hypoxemia (room air O_2_ saturation less than 90%).

**Table 3:** Diagnostic Criteria for Ileus

• No return of bowel function postoperatively. • Absence of passage of stool. • Absence of flatus. • Postoperative nausea and vomiting. • Abdominal distension. • Pathologic distension of the gastrointestinal tract radiographically. • Inability to tolerate discontinuation of nasogastric tube suction. • Inability to tolerate oral feeds. • Abdominal pain.

The six-hour period was developed by the empiric experience of one of the authors [B.C.W.] as being adequate to generate the desired outcome of decreased bowel distension. Patients had documentation of informed consent, physical examination, and radiologic confirmation of their diagnosis for entry into the trial. Radiologic investigation, preferably supine abdominal x-ray, was performed within 2 hours of completion of the oxygen dosing, as per standard of care.

This study was approved by the University of Florida Institutional Review Board.

We collected demographic data, the major illness underlying diagnosis of ileus, number of TOGA sessions, radiographic information before and after TOGA (maximal cecal diameter, maximal mid-transverse colon diameter, maximal mid-sigmoid colon diameter), complications, vital signs, and oximetry before and during TOGA. We assessed patients’ perceptions of TOGA.

Possible Discomforts and Risks: The use of NRB for delivery of oxygen is simple, safe, and inexpensive.^6^ In radiation pneumonitis, patients on NRB masks had a higher rate of intubation than those on high-flow nasal cannula.^9^ There are anecdotal reports of claustrophobia associated with oxygen mask use. Irritation of skin, nasal, or oral mucous membranes from the mask or the high flow of oxygen are possible. The mask could migrate cranially and irritate the eyes and adjacent structures. The sound of the high flow of oxygen may be disturbing to patients. If oxygen flow is interrupted, it is possible for subjects to become hypoxemic or hypercarbic while using NRB. If a patient were to vomit, aspiration pneumonia is possible. High concentrations of oxygen may cause lung injury or exacerbate hypercarbia in chronic lung disease.^10^

Possible Benefits: The subject may avoid risky medical interventions or invasive procedures if responsive to TOGA.

A paired-samples t-test was used to assess the difference in diameter between posttreatment and pretreatment. A *P*-value of less than .05 was considered statistically significant. Data analysis was conducted using SAS version 9.4 (SAS Institute, Cary, NC).

All authors/co-authors had access to the study data and had reviewed and approved the final manuscript. The ClinicalTrials.gov Identifier is NCT03386136.

## Results

**Figure 4:**
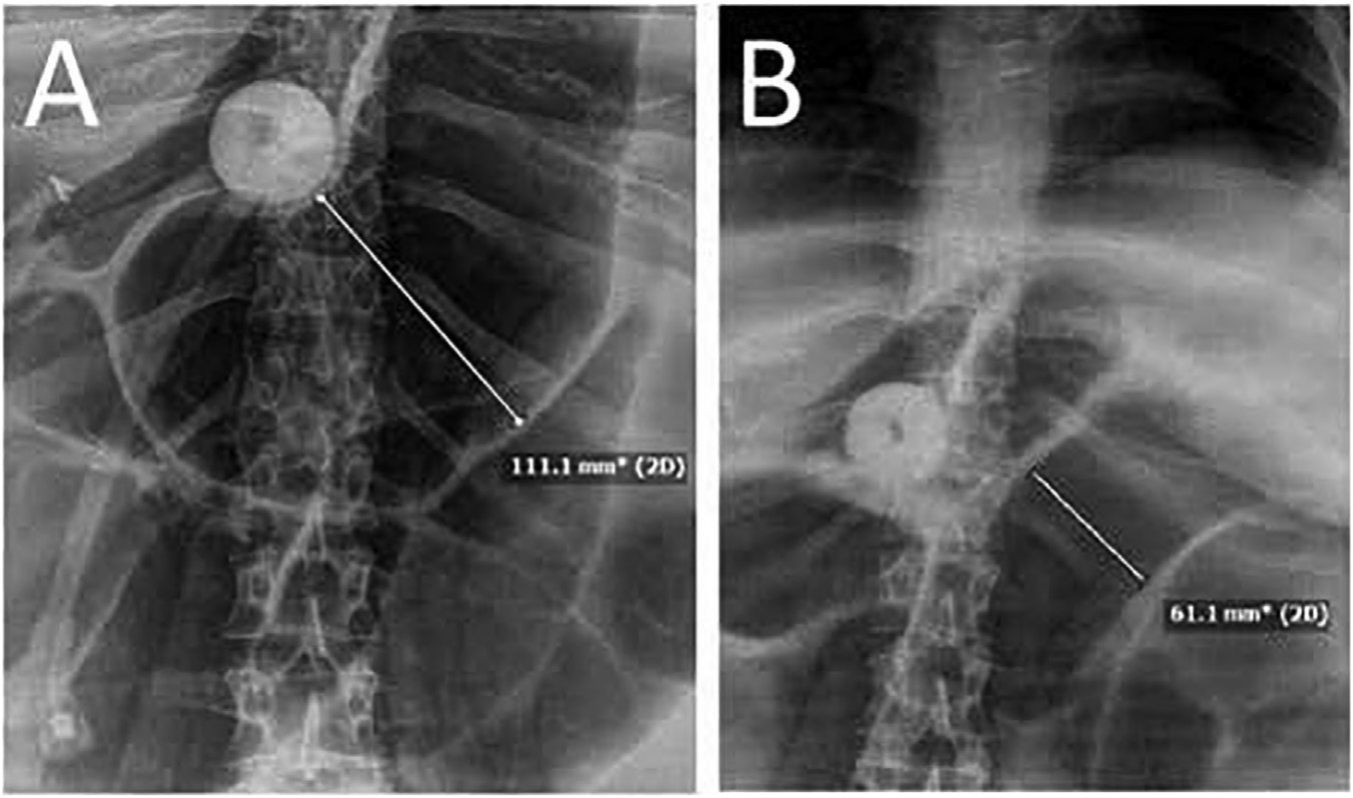
(A) There was a pre-TOGA treatment showing a small bowel diameter of 111 mm [normal ≤ 35 mm]. (B) After 6 hours of TOGA, the posttreatment diameter was 61 mm (55% of original diameter). There was a concomitant improvement in the patient’s symptoms.

Prior to initiation of the protocol, 3 patients were treated with standard informed consent. A middle-aged patient with a history of ulcerative colitis had status post total colectomy with end ileostomy. The patient was hospitalized on an inpatient gastroenterology service for refractory ileus, with gastroenterology faculty physicians serving as attendings. The patient did not respond to over 1 month of various treatments directed at ileus. There was a pre-TOGA treatment small bowel diameter of 111 mm [normal ≤ 35 mm]. After 6 hours of TOGA, the posttreatment diameter was 61 mm (55% of original diameter; see Figure 4). There was a concomitant improvement in symptoms.

In our preprotocol experience, there was a similar clinical response seen in a patient with colonic pseudo-obstruction, including improved appearance of roentgenographs. A bedridden nursing home patient had chronic constipation and a rectal diameter of 25 cm. After TOGA, there was no clear improvement in imaging, but the patient had his first spontaneous bowel movements in 5 years.

Data collection for our first eight protocol patients is shown in Table 4. The mean age was 53.6 ± 17.4 years. 62.5% were women. Diagnoses included postoperative ileus (POI), strangulated inguinal hernia, cystectomy, small bowel ileus (pancreatitis, sepsis), mechanical small bowel obstruction, and colonic ileus (sepsis). A clinical response was seen in 7/8 [87.5%] patients after TOGA. Patient 4, with malignant small bowel obstruction, failed to respond to TOGA. Patients 1 and 3 had colonic ileus. Others had small bowel ileus. All patients were held to nothing by mouth, had nasogastric tubes for venting, received intravenous fluids, and received parenteral proton pump inhibitors. Narcotics were not felt to be contributing to morbidity in these patients. 2 patients had bladder catheters. One patient received parenteral nutrition. No patients received neostigmine or alvimopan. No patients had endoscopic or surgical decompressions. Overall maximal lumen diameter in responders decreased by an average of 1.14 ± 0.87 cm [95% confidence interval = (0.34–1.95), *P*-value = .0131]. This was a 16% decrease in luminal diameter. In the colonic ileus, the maximal lumen diameter decreased by 1.6 ± 1.2 cm. In small bowel distension patients who responded to TOGA, the maximal lumen diameter decreased by 0.96 ± 0.54 cm. One patient had a complication possibly related to TOGA: transient tachycardia. There were no serious complications, such as bowel ischemia or perforation.

**Table 4:** Data From Pilot Study of 8 Participants

Patient	Age (y)	Sex	Initial lumen diameter (cm)	Posttreatment lumen diameter (cm)	Treatment outcome	Adverse reaction
1	25	M	11.5	11.1	Success	Yes
2	57	F	6.3	5.9	Success	No
3	57	M	15.8	13.0	Success	No
4	39	F	4.7	6.8	Failure	No
5	65	F	7.1	5.6	Success	No
6	80	M	6.1	4.6	Success	No
7	64	M	5.4	4.8	Success	No
8	42	M	6.5	5.7	Success	No

All these patients received 1 course of TOGA, except for patient 4, who received 2 courses. Even though improvements in measurements of bowel distension in aggregate were modest, success was defined as relief of obstipation. The decrease in bowel diameter was a surrogate marker for improved clinical condition.

In general, NRB was well tolerated. Other than transient tachycardia and 1 TOGA failure, all patients reported resolution of their ileus symptoms by the day after TOGA. Oximetry data and vital sign data were unremarkable and noncontributory.

## Discussion

In this study, we demonstrated that 100% oxygen administered by an NRB mask was a useful co-therapy in the medical management of bowel distension. This is the first clinical report of this novel TOGA technique. Oxygen therapy was well tolerated and did not interfere with other therapies administered to the patients.

In treatment of patients with distended bowels of any etiology, time is of the essence, as there are few treatments available for prolonged ileus. In mechanical bowel obstruction, delay in surgical treatment is associated with worsening clinical outcomes.^11^ In international consensus, prolonged POI was defined as obstipation on postoperative day 4 or after.^12^^,^^13^

Acute colonic pseudo-obstruction [ACPO], or Ogilvie syndrome, is distension of the colon with bowel gas due to an acute colonic motility disorder, or ileus. In a review of hospital records of 106,784 patients with ACPO, 45.7% had medical complications, 15.9% had procedural complications, and there was a mortality of 7.7%.^14^ Complications of ACPO include ischemia and perforation, especially with cecal diameter greater than 12 cm.^15^

Proposed treatment protocols for ileus and ACPO are directed at decreasing distension and restoring normal peristalsis.^16^, ^17^, ^18^ These include general supportive measures, pharmacologic measures, decompression with nasogastric tube, rectal tube, and/or colonoscopy, and surgery. Decompression aims at removing the gaseous and liquid contents of the bowel, decreasing distension of the bowel lumen. This would decrease bowel wall tension, allow improved circulation, and work cooperatively with other pharmacologic measures directed at improving peristalsis. Colonoscopic decompression can be provided in refractory cases. Surgery is reserved for the most severe cases or when there is an established perforation.

Even though NRB masks are purported to provide 100% oxygen to the patient, actual measurements show that the delivered percentage is less than 100%. The 3% formula (21% + oxygen flow rate in L/min × 3) is advocated as the best method to estimate FiO2.^19^ For the purposes of this discussion, we have used the estimate of delivery of 100% oxygen. Likewise, gas trapped in the bowel is not the exact composition of the ambient atmosphere. For illustrative purposes, we have estimated that gastrointestinal luminal air is 80% nitrogen and 20% oxygen.

Patients with severe underlying pulmonary disease were excluded from this study group. There is a concern that such patients, when presented with 100% oxygen, may become hypercarbic and deteriorate on that basis. In selected cases, it may be possible to provide short periods of high-flow oxygen via an endotracheal tube or noninvasive ventilation. Until further experience is available, such patients would need to be carefully selected and closely monitored. It may be worth considering a trial of TOGA in such patients, as they are at higher risk from other treatment interventions as well.^20^

Adverse consequences from the use of NRB are rare, including hypercapnia when oxygen flow is disrupted.^21^ Facial irritation and aspiration pneumonia are potential complications in up to 88% of selected populations.^22^

Hyperbaric oxygen for ileus has been described as far back as Fontaine in the late 19th century. Pneumatosis intestinalis has been treated with hyperbaric oxygen. More recently, hyperbaric oxygen therapy has been used effectively in those at risk of POI.^23^, ^24^, ^25^, ^26^ As a practical matter, the use of hyperbaric oxygen would be awkward to broadly apply to the systemically ill group of patients afflicted with colonic pseudo-obstruction, ileus, or bowel obstruction. Often, in these patients, clinicians must address acute systemic illness, body habitus, deconditioning, and delirium. These are all relative contraindications to hyperbaric oxygen therapy.

Oxygen toxicity (OT) has not been seen in TOGA. OT can manifest itself as inspiratory burning, cough, chest tightness, dyspnea, and reduced lung volume flows. OT is very rare in the time frames and barometric pressures contemplated by TOGA therapy, especially in under 24 hours.^27^ OT is the consequence of exposure of lung tissue to reactive oxygen and nitrogen species. In the hospital setting, this is confounded by coexisting pulmonary and cardiac pathologies. In healthy persons exposed to hyperoxia, as in divers, there are predictive equations to assess for OT. Consequences of OT are generally not seen at 40% FIO_2_ at 1 atmosphere. OT increases with time, barometric pressure, and level of physical activity. The POTindex is an algorithm that predicts OT in divers. TOGA exposure is calculated as 36, which is not consistent with toxicity (Arieli R, 2023, Personal communication).^28^

Physiologic POI may be exacerbated by narcotic use. In the ongoing effort to decrease hospital stays, same-day discharge after elective colectomy is being developed. In a recent review, 21% of such patients could be discharged.^29^ It is contemplated that TOGA, either in hospital or even at home, may contribute to efforts to treat ileus and allow early discharge after surgery. Narcotics inhibit resolution of ileus through activation of μ opioid receptors, inhibiting acetylcholine release from the myenteric nerve plexus. TOGA therapy, by decreasing luminal diameter, may facilitate myenteric plexus function and mitigate the need for narcotics and their adverse effects.^18^

High-flow oxygen has been successfully used, off-label, to treat headaches of various etiologies with uncertain mechanisms of action.^30^

The major strength of this study is the demonstration of the successful application of well-known physiology relating to the diffusion of gases across semipermeable membranes in a novel way. The effects are seen in a time frame that is clinically relevant. Some demonstrable effects are subjective, as in relief of malaise or passage of flatus. Some effects are objective, as in the decrease in intestinal luminal diameter. This was a relatively large effect. TOGA is plausible.

The major weakness of this study is its small size. This was an unblinded pilot study. There was no control group. Only a limited population was evaluated. This group was too small to make any statement regarding the potential for narcotic analgesics, often used in these settings, to counteract the therapeutic effects of TOGA.

Further research is needed to see if this TOGA effect is seen in other populations using a randomized design and placebo controls. A degree of ileus may be associated with almost every gastrointestinal problem leading to hospitalization. We will be looking to study the use of TOGA in these conditions. We will also be looking into prophylactic TOGA to decrease the length of stay following abdominal surgery.

## Conclusion

This is the initial report of a novel medical therapy for ileus, colon pseudo-obstruction, and bowel obstruction. TOGA provides periods of high concentrations of inspired oxygen to patients with sufficient lung capacity for gas exchange. This creates a gradient that drives nitrogen and other gases from the gastrointestinal tract. Resolution is accelerated by decreasing intestinal distension and restoring normal mechanical function to the distressed bowel wall. This was a pilot study to assess the feasibility of TOGA. It may also be applicable to the management of patients with gastroparesis and other functional bowel diseases such as irritable bowel syndrome, inflammatory bowel disease, and abdominal compartment syndrome. Larger studies are required to assess the efficacy of the provision of high concentrations of inspired oxygen in the early adjunctive medical support of all patients with ileus and bowel obstruction in conjunction with supportive therapy.
